# Dermatology

**Published:** 1897-09

**Authors:** Henry Waldo


### DERMATOLOGY.

**Ordinary baldness**, according to recent researches at the Hôpital St. Louis, by Dr. Sabouraud,[1] is a perfectly well characterised disease **due to specific micro-organism.** If his

[1] *Ann. de l'Inst. Pasteur*, 1897, xi. 134.

statements are found to be correct, the treatment of early baldness, or better still its prevention, should be a very satisfactory proceeding. He says that seborrhœa and alopecia areata are "essentially identical." The patch of alopecia areata is only an attack of acute circinated seborrhœa, and inversely the bald only become bald by a diffused process of chronic alopecia areata. "I understand well," he remarks, "how much this opinion will appear anarchic and monstrous to dermatologists, and, I believe, will be received by them with incredulity; but so did the statement that a boil and osteomyelitis are caused by the same organism meet with incredulity. The statement of every new fact is thus met."

There have been great differences of opinion as to whether alopecia areata, or what is known in France by the name of "pelade," is a contagious disease, and due to the growth of a vegetable parasite in the affected skin, or not. It has been noticed to spread in schools, and to be apparently conveyed to adults through contact with the lower animals; but in spite of this the neurotic theory to a large extent has held its ground. Dr. James Galloway says[1] that among British dermatologists Mr. Jonathan Hutchinson has proved that alopecia areata is a parasitic disease, and occurs among those who have previously suffered from tinea tonsurans, and that in 1891 Dr. Crocker[2] believed that one of the classes into which he divided the disease was due to the parasitism of a vegetable organism. At the February meeting of the Dermatological Society of London[3] Dr. Crocker brought forward an interesting case in support of his views. The patient, a young man, suffered from typical alopecia areata, and was one of ten patients who had developed the disease under the same conditions. At the same time Dr. Colcott Fox said that he had met with a similar epidemic of ten cases. Fifteen years ago Dr. Thin[4] stated that in the roots of hairs extracted from the margins of patches of alopecia areata, and between the root sheaths and the hair shafts, he had found bodies coinciding in appearance with bacteria, and he believed that alopecia areata was caused by the development in the hairs of a minute organism or bacterium. The objects described by Dr. Thin as organisms do not seem to have been situated exactly in the same position as the micro-bacillus so carefully studied by Sabouraud. He considered that the bacterium described by him as "decalvans" penetrated downwards between the internal root-sheath and the shaft towards the root of the hair. It then penetrated the hair-substance, and as it multiplied it proceeded upwards in the substance of the hair. Dr. Thin does not appear to have made any sections of the scalp, and did not succeed in staining the organisms, which renders an accurate comparison with such complete researches as Sabouraud's very difficult. Apart from the observations I

1 *Practitioner*, 1897, lviii. 519. 2 *Lancet*, 1891, i. 478, 533.
3 *Brit. J. Dermat.*, 1897, ix. 105. 4 *Brit. M. J.*, 1882, ii. 783, 828.

have mentioned there has been abundant clinical evidence that alopecia was a contagious disease. It is said that epidemics of the disease are by no means uncommon, especially in France. Alopecia areata has been noticed by Sabouraud to always start from a central point, and the baldness spreads from this centre by creeping in every direction along its circumference. He also saw that the most pathologically active zone of the patch was situated at the circumference, and that it is in this circumferential zone that the infected and broken hairs are found in the form of clubs. According to Sabouraud's histological facts the micro-organism resides in the active peripheral zone, and more exactly in the dilated orifices of the hair follicles. This micro-organism, which he constantly found, is a small bacillus infesting the upper part of the hair-sac. He also noticed that the orifices of the follicles were filled with a fatty substance which could be squeezed out on pressure, and which he called the "seborrhœic cocoon." In this seborrhœic cocoon could be found, surrounded by a crowd of other microbes, the same organism which he demonstrated in the histological sections. He then set about isolating this bacillus, and cultivating it, and trying to prove that it is the actual cause of alopecia areata. It was necessary to discover a medium which should have the power of destroying all micro-organisms except the one in question. This preparation consists of a very acid culture medium. The ingredients are the following:

| | | | |
|---|---|---|---|
| Peptone... ... | 20 grammes | Water ... | 1000 grammes. |
| Glycerine ... | 20 ,, | Gelose ... | 13 ,, |
| Acetic acid ... | 5 drops | | |

With this medium one obtains in many of the tubes, in the midst of other colonies, one or two pure cultures from the beginning, which are visible on the third to the fourth day, the temperature being 35° C. This is the most favourable temperature, but it grows at 39° C., a temperature quite incompatible with the trichophyta. They show as pointed mounds, the colour of which is pale at first, but they gradually acquire a very characteristic brick-red. This brick-red tint of the microbe is most distinct. It is a superficial growth, and takes no firm hold of the underlying soil. A series of researches was then undertaken which resulted eventually in the demonstration of the microbial nature of baldness. A preliminary study of seborrhœa in the hairy scalp revealed: (1) that the bacillus of the brick-red cultures from alopecia areata is also present in the seborrhœic plugs of the mouths of the hair and sebaceous follicles in seborrhœa, that it is there present in considerable quantity, and that it is certainly the cause of seborrhœa; (2) that consequently seborrhœa and alopecia areata have a common origin from the same micro-organism. Finally, having studied the obvious relations existing between seborrhœa and the habitual falling out of hair, Sabouraud came to the conclusion that the disseminated loss of hair in seborrhœa was the prelude of baldness. It will be interesting to see what Dr. Unna says to all this, as his pathology of seborrhœa and Sabouraud's are certainly at variance. The histology of bald scalps shows that the mechanism of the process leading to baldness is as follows: Whenever the specific bacillus of seborrhœa invades a follicle, it produces around it, and especially at its base around the hair-papilla, an afflux of wandering cells. The papilla gradually atrophies, producing as it does so a hair which is progressively more and more frail and devoid of pigment. Finally, although very slowly, it dies, and the dead hair is expelled. But before the death of the hair-papilla it makes several attempts at regeneration. In this seborrhœic infection of the hairy scalp the colonies of bacilli are enormously abundant; the sebum which is effused on the surface of the skin in the form of an apparently homogeneous crust, is composed of an infinite number of seborrhœic plugs turned out of the follicles, and each of these plugs contains the bacillus in millions. In hairy scalps which have been once invaded, the microbial infection remains endemic and settled, so that a hair once shed is never renewed. Furthermore, the permanent effusion of this germ-bearing sebum infects one by one the follicles which have remained sterile. In this way ordinary baldness is little by little established; the progressive sclerosis of all the elements of the hair follicle brings with it considerable changes of form. The whole part of the follicle invaded by the bacterial colony becomes hollow and broken up by narrow diaphragms, which render the seat of infection inaccessible to external antisepsis. The follicles may ultimately be converted into fibrous cords. But the incredible abundance and absolute purity of the infection persist, even when the baldness is finally and definitely established. Even at this terminal stage of its evolution, ordinary baldness remains the most abundantly and most purely microbic malady known in the skin. Such are the conclusions to which clinical and histological results lead the author; but a further difficulty has to be cleared up. It is that at the moment of infection the bacterial colony does not invade the hair-papilla, but remains in the upper third of the follicle. By what mechanism can the colony exert its influence upon the papilla? One can only admit an action at a distance, and this remains to be proved. Sabouraud, thinking then that this distant action was effected by the toxins of the micro-organism, devised and carried out the following experiment, which is conclusive and practically seals this chapter on the microbial nature of baldness. He made a cultivation on a liquid medium, and having filtered it through porcelain, inoculated the filtrate deeply under the skin and into the muscular tissues of a rabbit. The rabbit at once commenced to shed its fur, and within forty days from the date of inoculation general alopecia was established. This experiment is of the greatest value as shewing that the toxin of the bacillus of seborrhœic plugs is so specific and individual that when inoculated into the heart of the system it retains its elective and exclusive action on the papilla of the cutaneous hairs. As this bears a most important relation to the treatment of tinea tonsurans, I must add that after four years devoted to the mycological and therapeutical study of this intractable and often incurable disease, Sabouraud came to the conclusion that no antiseptic treatment is absolutely efficacious in the case of tinea fungus which has invaded the hair as far as the root. This is due to the inaccessibility of the root to antiseptics, owing to the narrowness and depth of the hair follicle, which prevent the diffusion of all the therapeutic agents which have been employed. He has, furthermore, become convinced that if, as appears in favus, one could make a complete series of epilations without breaking the hairs, tinea tonsurans could be rapidly cured. But the diseased hairs break off close to the surface, and so it is necessary to take care to enucleate them by working somehow from below, by the root or even the follicle, killing the latter "temporarily." Following out this line of argument and applying it to alopecia areata, Sabouraud inferred that if the loss of hair in this disease is due to microbial intoxication, the toxins of the micro-organisms would be capable of inducing alopecia. It would then be possible to use them to produce a temporarily bald area at any desired spot, and so to cause a spontaneous falling-out of the tinea-infected hairs, which are too fragile to be removed by epilation.[1] So that the treatment of tinea tonsurans is likely to be revolutionised. One little difficulty I must mention here. It is, that the specimen of bacilli cultivated from "pelade" reproduces bald patches, like alopecia areata, in the sheep, guinea pig, and rabbit; the other specimen, derived from oily seborrhœa, fails to cause in these animals the slightest sign of seborrhœic alopecia. For the treatment of ordinary baldness, Sabouraud suggests that three times a week, in the evening, there should be applied to the affected skin a pomade containing sulphur, oil of cade, and yellow oxide of mercury. On the following morning the skin is to be thoroughly washed with soap, and subsequently a solution containing equal parts of alcohol and ether, in which resorcin has been dissolved to the extent of two per cent., should be applied by means of a brush.[2] It will have been noticed that Sabouraud's culture-medium was a very acid one, and on that account I sometimes order for cases of seborrhœa an alkaline preparation, which seems to answer very well, of which the ingredients are the following:

| | |
|---|---|
| Thymol, ʒj. | Glycer., ʒiv. |
| Liq. Potassæ, ʒj. | Aq. Sambuc., ad ℥viij. |

Long ago Unna called attention to the efficacy of resorcin

[1] For this summary of Dr. Sabouraud's work I am largely indebted to a paper by Dr. L. Wickham (*Brit. M. J.*, 1897, i. 1028) and to a report by Dr. Leslie Roberts (*Brit. J. Dermat.*, 1897, ix. 219).

[2] *Practitioner*, 1897, lviii. 523.

in cases of **seborrhœa**; it can be used as a watery solution up to twenty per cent. The following, as suggested by Dr. Crocker, will be found useful and agreeable:

| | |
|---|---|
| Ac. Acetic, ℥ss. | Eau de Cologne, ℥ij. |
| Resorcin, ʒj. | Aq. Ros., ad ℥viij. |

Add a little glycerine if the hair is very dry. Rub well in, crown and temple especially, once daily for six to eight weeks. Or I sometimes use chinosol, 1 in 200 of water; or if a pomade is preferred, chinosol 2 parts, distilled water 2 parts, vaseline 26 parts. A remedy suggested by Dr. J. F. Payne[1] is glycerine of tannic acid. He says it has an undoubted effect on the scurf of the head. Its exact mode of action, he says, is not easy to explain. But he believes the only thing which permanently succeeds in stopping this kind of affection is, broadly speaking, absolute disinfection or sterilisation of the scalp. It is a fact, Dr. Payne says, familiar to surgeons, that wounds of, or operations on, the scalp have always been regarded as more dangerous, more liable to give rise to unhealthy suppuration and pyæmia, than those of other parts of the body, the reason being that this part of the surface is swarming with micrococci of the kind which produce suppuration. Payne would treat the scalp thoroughly for three days with perchloride of mercury solution, 1 in 1000, or, if the skin is a delicate one, 1 in 2000. But this treatment is not enough. Although the condition appears to be improved, it is apt to return, and irritation is often produced by the antiseptic. Something seems to be required to improve the nutrition of the scalp as well as to destroy superficially the germs that may be there. The next remedy to apply is one undoubtedly useful in all sebaceous affections, namely, sulphur, which has a most definite effect. Considering that we also want an antiseptic, the following is useful: Sulph. precip. gr. xv., ac. carbol. ♏xv., vaseline (or some excipient) ℥j.; to which may be added two or three drops of oil of lavender, or oil of bergamot, or any other scent. Coal tar solution or creasote may be substituted, if preferred, for the carbolic acid. When this has been used for a week or two the effect on the scalp is extraordinary. The scurf disappears almost entirely, and the condition of the hair is improved. Or I sometimes order a more elegant preparation of sulphur; namely, Behrendorf's "pomade soufrée" (May, Roberts & Co.) which Ferris & Co. procure in collapsible tubes. Dr. Thin rubs in ung. sulphuris or lin. terebinthinæ to the edge of a patch of alopecia, he says with great success. The same applications may be used for seborrhœa. Dr. Payne says that washing and pure antiseptic treatment should be repeated from time to time, say once a week. At the same time, brushes, combs, hat linings, &c., should also receive antiseptic treatment, or be destroyed. After a course of treatment such as this, Payne thinks, it will generally be desirable to restore to the hair some

[1] *Clin. J.*, 1894, iii. 105, 121.

of the oily matter which it has lost. It may be done by rubbing in oil, or, still better, a soft pomade made with beef-marrow (which must be clarified). But most patients, especially ladies, object to any kind of greasy application to the hair. In many of these conditions of the hair and skin there is bad nutrition generally. The skin may look rather coarse and badly nourished. In all such conditions, independent of anything which can be called disease, Payne considers that arsenic internally is very useful. It improves the complexion even of people suffering from no obvious disease; and it improves the nutrition of the skin in cachectic or anæmic conditions.

HENRY WALDO.

